# GWAS *loci* associated with Chagas cardiomyopathy influences DNA methylation levels

**DOI:** 10.1371/journal.pntd.0009874

**Published:** 2021-10-29

**Authors:** Desiré Casares-Marfil, Martin Kerick, Eduardo Andrés-León, Pau Bosch-Nicolau, Israel Molina, Chagas Genetics CYTED Network, Javier Martin, Marialbert Acosta-Herrera

**Affiliations:** 1 Institute of Parasitology and Biomedicine López-Neyra, CSIC, Granada, Spain; 2 Unidad de Medicina Tropical y Salud Internacional Hospital Universitari Vall d’Hebron, PROSICS, Barcelona, Spain; Instituto de Investigaciones Biotecnológicas, ARGENTINA

## Abstract

A recent genome-wide association study (GWAS) identified a locus in chromosome 11 associated with the chronic cardiac form of Chagas disease. Here we aimed to elucidate the potential functional mechanism underlying this genetic association by analyzing the correlation among single nucleotide polymorphisms (SNPs) and DNA methylation (DNAm) levels as *cis* methylation quantitative trait *loci* (*cis*-mQTL) within this region. A total of 2,611 SNPs were tested against 2,647 DNAm sites, in a subset of 37 chronic Chagas cardiomyopathy patients and 20 asymptomatic individuals from the GWAS. We identified 6,958 significant *cis*-mQTLs (False Discovery Rate [FDR]<0.05) at 1 Mb each side of the GWAS leading variant, where six of them potentially modulate the expression of the *SAC3D1* gene, the reported gene in the previous GWAS. In addition, a total of 268 *cis*-mQTLs showed differential methylation between chronic Chagas cardiomyopathy patients and asymptomatic individuals. The most significant *cis*-mQTLs mapped in the gene bodies of *POLA2* (FDR = 1.04x10^-11^), *PLAAT3* (FDR = 7.22x10^-03^), and *CCDC88B* (FDR = 1.89x10^-02^) that have been associated with cardiovascular and hematological traits in previous studies. One of the most relevant interactions correlated with hypermethylation of *CCDC88B*. This gene is involved in the inflammatory response, and its methylation and expression levels have been previously reported in Chagas cardiomyopathy. Our findings support the functional relevance of the previously associated genomic region, highlighting the regulation of novel genes that could play a role in the chronic cardiac form of the disease.

## Author summary

Genome-wide association studies (GWAS) have provided extensive information regarding the genetic component of complex traits, including parasitic diseases such as Chagas disease. However, these associations mapped in regulatory regions of the genome and assigning them a functional consequence have been cumbersome. In this study we aimed to evaluate the functional mechanism underlying the previously reported genomic association with chronic Chagas cardiomyopathy, by assessing the correlation between methylation changes and the underlying genetic variations within the region. These methylation quantitative trait *loci* (mQTLs) may be involved in gene expression regulation. We identified mQTLs in three genes that have been associated with cardiovascular diseases in previous studies. Interestingly, one of these genes was previously identified as differentially methylated and expressed in heart biopsies of chronic Chagas cardiomyopathy patients. Our results suggest novel genes that could play a role in the chronic Chagas cardiomyopathy, evidencing the functional relevance of the previously associated *loci*.

## 1. Introduction

Chagas disease is a parasitic infection caused by the protozoan *Trypanosoma cruzi* that affects ~7 million people around the world and is endemic in Latin America (https://www.who.int/health-topics/chagas-disease#tab=tab_1). After the contact with the parasite, infected individuals develop an acute phase followed by an indeterminate phase where they remain asymptomatic in the majority of the cases [1]. Nevertheless, around 30% of Chagas’ disease patients develop a chronic phase with cardiac involvement known as chronic Chagas cardiomyopathy, which is the most severe form of the disease characterized by inflammation of the cardiac tissue [1,2].

Given the existence of differential susceptibility to Chagas disease and in the development of its chronic cardiac form, several genetic studies have been performed in order to elucidate genetic variation associated with disease risk [3]. A recent genome-wide association study (GWAS) carried out in Latin American populations identified a significant association with the chronic cardiac form located at ~6.4Kb downstream of the transcription start site of the *SAC3D1* gene, in the region q13.1 of chromosome 11 [4]. Moreover, several other genes within this locus were previously related with cardiovascular traits and were found to have functional relevance in the disease [4]. As in other complex diseases, the associated and suggestive variants mapped in non-coding regions of the genome [5,6], being these well-known to exert their effect through modulating gene expression [7]. The DNA methylation (DNAm) of a particular locus might be driving this modulation through methylation quantitative trait *loci* (mQTLs), which are correlations among single-nucleotide polymorphisms (SNPs) and DNAm levels in the context of a specific trait [8].

In order to identify the functional effect of the SNPs within the previously associated region, we conducted an analysis of mQTLs acting on nearby methylation sites (*cis*-mQTLs) integrating this genetic variation with DNAm levels within a 2 Mb window, in a subset of cardiomyopathic and asymptomatic Chagas disease patients included in the GWAS. This assessment will contribute to the functional characterization of the associated *loci*, and the identification of variation in the methylation patterns of chronic Chagas cardiomyopathy patients driven by genetic variation. This will provide novel insights into the pathogenesis of the disease.

## 2. Material and methods

### 2.1. Ethics statement

This study was approved by the Ethics Committee from the Vall D’Hebron University Hospital, Barcelona, Spain (PR (AMI) 297/2016). The Ethics Committees for the GWAS data was described elsewhere [4]. Protocols used in the study followed the principles of the Declaration of Helsinki and all individuals included in the study signed written informed consents.

### 2.2. Study samples and ethical considerations

All donors were recruited by the health care system from the Vall D’Hebron University Hospital, Barcelona, Spain. The study population is composed by a subset of patients from Bolivia from the previous GWAS [4]. A total of 57 seropositive individuals for *T*. *cruzi* antigens were classified into chronic Chagas cardiomyopathy patients (cases, n = 37) and asymptomatic (controls, n = 20) according to the presence of cardiac abnormalities. Patients were subjected to electrocardiogram and echocardiogram tests to determinate cardiac abnormalities, while additional clinical information or complementary tests were retrieved from the medical history. Case-control sample size and demographic information are summarized in Table 1.

**Table 1:** Demographical characteristics and sample size.

	CCC	ASY
**Subjects, N**	37	20
**Sex (% females)**	18 (48.65%)	16 (80%)
**Age (Mean±SD)**	52.8±9.9	45.5±12.9

The sample sizes refer to data passing the genotyping quality controls. Abbreviations: CCC, chronic chagas Cardiomyopathy; ASY, asymptomatic.

### 2.3. Data preparation

#### 2.3.1. SNP genotyping and imputation

As extensively described elsewhere [4], blood DNA was isolated and genotyped using the Global Screening Array Platform (Illumina Inc., San Diego, CA, USA). After passing their corresponding QCs using the software PLINK v.1.9 [9], genotyped data was imputed with the Michigan Imputation Server [10].

After imputation, genomic information from the 2 Mb flanking region centered in the variant associated with the disease (rs2458298, chr11:63814813–65814813, according to build hg19) was extracted from a subset of individuals from the Bolivian cohort, and transformed to PLINK format. Imputed SNPs were filtered by their imputation quality metric Rsq and MAF, keeping for the analysis those that satisfied both MAF>5% and Rsq>0.3.

#### 2.3.2. DNA methylation data preprocessing

Genomic DNA from blood samples from the 57 Bolivian individuals was isolated using the QIAamp MidiDNA Kit (QIAGEN, Germany) following manufacturer’s recommendations. From this, methylation patterns were determined using the Infinium MethylationEPIC Bead Chip array (Illumina, Inc., San Diego, CA, USA). A total of 850,000 DNAm sites were assessed and passed to downstream data processing and normalization using the R/Bioconductor package *minfi* [11]. Probes positioned in SNP positions and those with a detection *p-value*<0.01 were removed. The clustering of methylated and unmethylated probes did not show significant differences in the samples. After this, the preprocessQuantile method for normalization was performed [12] and the *M* values matrix was obtained by the log_2_-transformation of the DNA methylation ratio. This matrix was utilized to compare sample groups (cardiomyopathic and asymptomatic) by an eBayes-moderated paired t test using the limma package [13]. As for the genomic data, methylation data was extracted for the same 2 Mb region centered in the variant associated with the disease and *p-value* of < 0.05 was considered statistically significant.

### 2.4. Methylation quantitative trait loci statistical analysis

In order to link genetic variants to variations in DNAm, preprocessed methylation and imputed genotyped data from the significant region were integrated for the 57 individuals included in this study. The analysis of *cis*-mQTLs for cases and controls was performed using the R/Bioconductor package *MatrixEQTL* version 2.3 [14]. For the correlation analyses, sex and age were used as covariates and those SNP-DNAm site pairs with a maximum distance of 1 Mb were tested. In order to assess the functionality of the locus associated with the cardiac form of Chagas disease, the integration analysis was carried out in the region of 2 Mb centered in the associated GWAS variant (rs2458298, chr11:63814813–65814813 according to build hg19). Those interactions with a False discovery rates (FDR) of <0.05 were considered as significant.

### 2.5. In silico functional analyses

An *in silico* functional analysis was performed to assess the biological consequences of the genes related with the leading significant interactions. For this, genes within the DNAm sites were annotated using the R/Bioconductor package IlluminaHumanMethylationEPICmanifest (https://bioconductor.org/packages/release/data/annotation/html/IlluminaHumanMethylationEPICmanifest.html).

In order to prioritize *cis*-mQTLs linked to the variant associated with the disease, the LD link tool [15] was used to calculate SNPs in LD (r^2^>0.4) with it in the admixed American population from The 1000Genomes Project (1KGP). The Open Targets Genetics web tool (https://genetics.opentargets.org/) was used to evaluate the biological and disease implications of the genes associated with the DNAm sites from the prioritized *cis*-mQTLs.

## 3. Results

### 3.1. Identification of cis-mQTLs in the previously associated chronic Chagas cardiomyopathy locus

**Fig 1:**
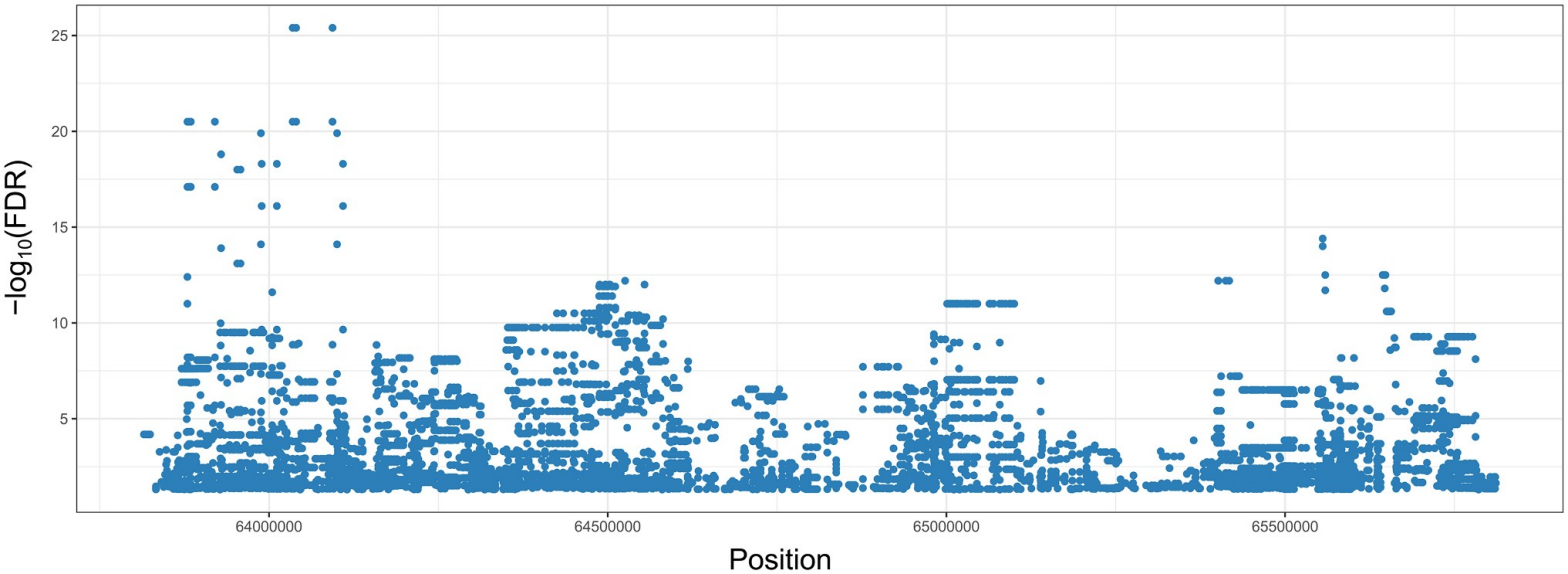
Regional scatter plot of the *cis*-mQTL results for the selected genomic region under analysis (chr11:63814813–65814813). Representation of the 6,958 *cis*-mQTLs identified in the genomic region previously associated to the disease. The x-axis corresponds to the genomic position of the SNP from each mQTL interaction; the y-axis refers to the transformed False Discovery Rate (FDR) of each signal.

Genomic and methylation data from a total of 57 Chagas disease patients were integrated to determine *cis*-mQTLs. We limited our analysis to the genomic region centered in the variant previously associated with the chronic cardiac form of the disease [4]. A total of 2,611 SNPs were tested against 2,647 DNAm sites in 37 chronic Chagas cardiomyopathy patients and 20 asymptomatic individuals after passing their corresponding quality control (QC) criteria. We identified 6,958 significant *cis*-mQTLs (FDR<0.05) at maximal 1 Mb distance of the GWAS leading variant (Fig 1). These *cis*-mQTLs were composed by 2,143 unique SNPs and 152 unique DNAm sites. On average, the distance between the SNPs and DNAm sites that form the *cis*-mQTLs was ~114 kb.

First, we evaluated if the chronic Chagas cardiomyopathy associated variant and those in linkage disequilibrium (LD) acted as *cis*-mQTLs. We identified six SNPs with moderate LD (mean r^2^ = 0.55) with the leading variant as *cis*-mQTLs of a DNAm site located between 200–1,500 bp of distance to the transcription start site (TSS1500) of the SAC3 Domain Containing 1 (*SAC3D1*) gene (S1 Table). The risk alleles of these variants were associated with higher methylation levels (S1 Fig). However, these changes were not significant when comparing the methylation status in asymptomatic and cardiomyopathic patients. Nevertheless we observe a highly significant positive correlation of GWAS and *cis*-mQTL p-values (S2 Fig) suggesting an association of both methylation and genetic signals. The most significant *cis*-mQTL in the region, which mapped in the gene body of the Phospholipase C Beta 3 (*PLCB3*), also showed no significant methylation changes. Therefore, although we observe a significant correlation of the allele changes with the methylation levels, we cannot reassure that this is directly related to the disease as this alteration in methylation levels occurs in a similar manner in asymptomatic and CCC patients.

**Fig 2:**
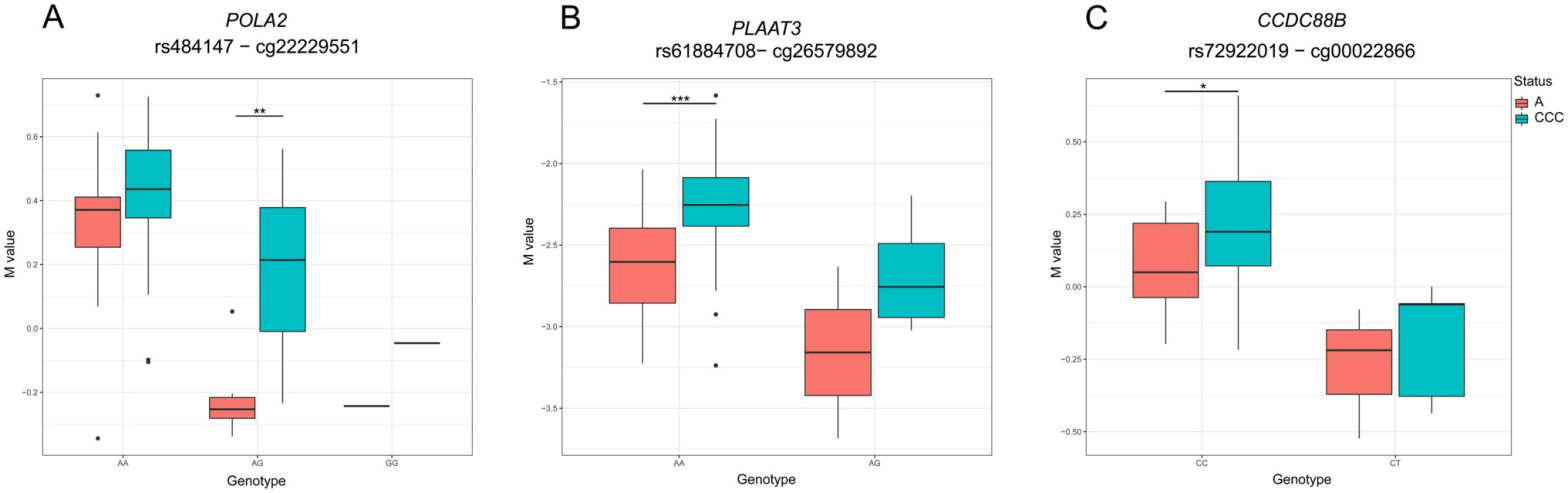
Box plots of three of the most interesting genes comparing DNAm levels between chronic Chagas cardiomyopathy patients (CCC) and asymptomatic individuals (A). Comparison of individuals’ SNP genotypes and CpGs Mvalues for the most interesting mQTL genes that also showed differences in methylation patterns in cases and controls. Significant differences between genotypes are marked with symbols according to their level of significance calculated with an ANOVA test (* *p-value*≤0.05; ** *p-value*≤0.01; *** *p-value*≤0.001).

Second, taking into account the total *cis*-mQTLs identified in the evaluated region, we found 268 interactions with significant methylation changes when comparing asymptomatic individuals and chronic Chagas cardiomyopathy patients. 89 (33%) DNAm sites were located in promotors (distance to TSS < 1500 bp), 175 (65%) in gene-bodies and 4 (1%) in intergenic regions (Table 2). The majority of these interactions affected a DNAm site located in the gene body of the DNA polymerase alpha 2, accessory subunit (*POLA2*) (Fig 2A) and also contain the most significant *cis*-mQTL in this locus (11:65063553−cg22229551, FDR = 1.04x10^-11^, R^2^ = 0.72). Additionally nine SNPs from these *cis*-mQTLs were nominally associated with the disease in the previous GWAS [4].

**Table 2:** *cis*-mQTLs interactions that produce differential methylation among chronic Chagas cardiomyopathy patients and asymptomatic individuals.

SNP ID	DNAm site ID^a^	Distance (bp)^b^	DNAm site function^c^	DNAm site gene	mQTL *p-value*	mQTL FDR	Beta	R^2^^d^
rs72922019	cg00022866	-104,203	Gene body	*CCDC88B*	1.58E-05	1.89E-02	-0.37	0.30
rs1111934	cg14293362	450,196	TSS200	*TM7SF2*	3.35E-05	3.38E-02	-0.30	0.28
rs12292693	cg19940438	-146	TSS1500	*SPDYC*	1.24E-10	7.75E-07	0.67	0.55
rs12292693	cg16849481	-260	TSS1500	*SPDYC*	2.93E-08	9.43E-05	0.47	0.45
rs484147	cg22229551	-241,058	Gene body	*POLA2*	8.78E-06	1.18E-02	-0.30	0.32
rs61884708	cg26579892	478,276	Gene body	*PLA2G16*	4.80E-06	7.22E-03	-0.60	0.33
rs507062	cg23228688	69,522	Intergenic	*-*	2.01E-05	2.28E-02	-0.71	0.30

^a^DNAm site identificator

^b^SNP-DNAm site genetic distance.

^c^Position of the DNAm site respecting its annotated gene. TSS1500 and TSS200 refers a position at 1500-200 or less than 200 bp to the gene promoter.

^d^mQTL correlation.

The *cis*-mQTL with the most significant variation in the methylation patterns among cases and controls (*p-value* = 8.09x10^-04^), corresponded to the DNAm site within in the gene body of the Phospholipase A and acyltransferase 3 (*PLAAT3*) gene (rs61884708−cg26579892, FDR = 7.22x10^-03^, beta = 0.12, R^2^ = 0.33, Fig 2B). Among these results it is interesting also to highlight the interactions with the DNAm site located in the coiled-coil domain containing 88B (*CCDC88B*) gene body (rs72922019 − cg00022866, FDR = 1.89x10^-02^, beta = 0.08, R^2^ = 0.30, Fig 2C). This gene was previously reported to be differentially methylated and differentially expressed when comparing biopsies from heart tissue of chronic Chagas cardiomyopathy patients and healthy donors [16], and we were able to corroborate these findings in an independent population.

### 3.2. Functional annotation analysis

To explore functional features of the genes related to the identified DNAm sites, several databases and online tools were queried. The majority of the methylated positions in the region were located within gene bodies (S3 Fig), and those *cis*-mQTLs that interacted with DNAm sites located in intergenic regions were removed from further functional analyses.

According to the Open Targets Genetics database there are several association studies that have related the *POLA2* with hematological traits, such as mean corpuscular hemoglobin or red blood cell count [17,18]. One of these studies has also found an association of this locus with cardiovascular disease (*p-value* = 6x10^-16^), highlighting the role of this gene in this trait [17]. Additionally, different *loci* in the *CCDC88B* gene have been also associated with cardiovascular disease in the same dataset (*p-value* = 1x10^-10^) [17]. Taking together, the *in silico* functional analysis of the resulting genes suggests the relationship of the associated region with different cardiovascular traits, supporting its relevance in the pathogenesis of the chronic Chagas cardiomyopathy.

## 4. Discussion

In this study we conducted the first *cis*-mQTL analysis in Chagas disease patients from the Bolivian population, leveraging a previously reported genomic association, and its correlation with specific DNAm levels that may impact the regulation of gene expression. This assessment has provided further functional characterization of the associated locus, and has revealed significant interactions with relevant genes that have been previously related with several cardiovascular traits. The chronic cardiac form of the disease shows damage and inflammation of the myocardium as a response to the parasite, but also the microvascular system can be involved [19]. Interestingly, our results revealed the regulation of gene expression of an inflammation related gene, validating previous findings, and highlighting the use of whole blood as a surrogate tissue for studying the chronic form of the disease. In addition, we identified regulatory mechanisms potentially involved on disease pathogenesis, exposing the functional implication of the previously associated *loci*.

Considering the sustained host-parasite interaction throughout human history, host genetic variation has been linked to the heterogeneous response observed to infectious agents [20]. Nevertheless, functional studies are needed to determine the relevance of this genetic variation on disease pathogenesis [8]. In this sense, epigenomic modifications are particularly ligated to pathological processes behind the chronic phases of infections, even when the first stages have been overcome [21,22]. Several of these modifications have been described in infectious diseases like viral [23], bacterial [24] and specifically, DNAm changes has been reported in parasitic diseases, such as Malaria, Leishmaniasis and Chagas disease [16,25].

The present work showed *cis*-mQTLs composed by SNPs in LD with the leading GWAS variant that were correlated with changes in the DNAm levels in the vicinity of the promoter of *SAC3D1*. This gene, also known as *SHD1*, is a transcriptional regulator of STAT5 [26], and increased phosphorylation levels of this protein have been associated with protection in human hearts [27]. Among the significant interactions identified in the region, the top signal corresponded to a DNAm site located in the gene *PLCB3*, which encodes a member of the phospholipase C family. This protein family has been described to act in the induction of cardiac hypertrophy [28,29]. Moreover, another member of the phospholipase C family, the *PLCB2*, showed a significant correlation between methylation and gene expression levels in cardiac tissue, compared to healthy donors in a previous study in chronic Chagas cardiomyopathy [16]. Although our results do not show significant differences on the methylation levels among asymptomatic and cardiomyopathic patients, we might speculate their potential role on the disease, based on their localization within the GWAS locus and their functional relevance.

From the total number of significant *cis*-mQTLs it is relevant to highlight those that produce substantial changes in methylation levels when comparing patients with chronic Chagas cardiomyopathy and asymptomatic individuals, since these interactions could have a greater implication on the regulation of gene expression in the pathogenesis of the disease. From these, the *CCDC88B* gene is of special interest because of its involvement in inflammatory conditions [30]. This gene showed higher methylation levels within the gene body in cardiomyopathy patients, which is related with tissue-dependent regulation of gene expression [31]. The *CCDC88B* has been associated with the most severe form of Malaria, cerebral malaria, where patients suffer inflammation of the brain tissue [32]. In this study, the authors tested the role of the *CCDC88B* during this affection in a murine model and showed that is a potential regulator of T cells function. This gene plays an important role in the inflammatory and host responses during parasitic infections [32,33]. This is of substantial interest since inflammation of the cardiac tissue also occurs during chronic Chagas cardiomyopathy [19,34]. In addition, *CCDC88B* has been also described in a murine model as relevant in the immune function of dendritic cells [30]. In this sense, one subtype of dendritic cells, the tolerogenic dendritic cells, have been recently proposed to have therapeutic properties in a mouse model of chronic Chagas cardiomyopathy, given their role in the reduction of inflammation and fibrosis that contributes to reduce disease progression [35,36]. Interestingly, the *CCDC88B* was previously reported to suffer changes in its methylation and expression levels when cardiac tissues from patients with chronic Chagas cardiomyopathy and healthy donors were compared, showing also a correlation among both methylation an expression variations [16]. The identification of this gene in whole blood from independent study samples highlights its role in the pathogenesis of the chronic Chagas cardiomyopathy, as well as emphasize the use of whole blood as a surrogate tissue that allows the detection of disease-related molecules in a less invasive way. Thus, despite the high tissue specificity of the DNAm patterns [37], surrogate tissues as whole blood could be widely used, as in other diseases with high correlation of results [38].

The *PLAAT3* gene, also known as *PLA2G16*, showed the most significant variation in methylation levels, being also the chronic Chagas cardiomyopathy patients’ methylation levels higher in this locus in comparison with asymptomatic individuals. This gene encodes an adipose-specific phospholipase whose expression has been reported to be reduced in patients with peripheral artery disease, which is a common affection in patients with coronary artery disease [39,40]. Thus, as mentioned for *PLCB3*, the role of the phospholipases during *T*. *cruzi* infection has been widely studied as part of the lipid metabolism [41]. Nevertheless, although this phospholipase seems to be related with other cardiovascular traits and exhibit higher expression in heart tissue, its potential relation with Chagas disease remains to be determined.

Finally, the most significant *cis*-mQTLs that produced variation in the methylation levels among cases and controls corresponded to DNAm sites located in the gene body of the *POLA2*. As for the other genes, *POLA2* showed higher methylation levels in chronic Chagas cardiomyopathy patients. Our *in silico* functional analyses related this gene with different hematological measurements in other association studies, in addition to be related with cardiovascular disease [17,18]. One of the hematological traits associated with *POLA2* is the mean corpuscular volume, which has been previously related with heart failure [42]. In this sense, the relation of this gene with the pathogenesis of the chronic Chagas cardiomyopathy remains unclear, as gene expression information would be necessary to verify the direction of the reported methylation patterns. However, the identification of genes previously related to several cardiovascular traits and cardiac damage reinforces the role of the region under study previously associated with the disease [4]. Therefore, our results suggest the DNAm as a possible driver of the variation observed in the differential development of the chronic Chagas cardiomyopathy.

Our study has some limitations that are worth mentioning here. First, as the variation explained by *cis*-mQTLs is higher in blood [43], we focused our work in these interactions. Nevertheless, the addition of genetic distal interactions (i.e. *trans*-mQTLs) to the analysis could be also informative about other genes and pathways relevant for the disease. Interestingly, we used whole blood as a surrogate tissue and were able to validate the involvement of a previously reported gene in the cardiomyopathy. Further studies on cardiac tissue will be relevant to validate our findings, as well as functional analysis to decipher the specific mechanisms by which the SNP modifies methylation levels. Another limitation is the analysis of variants with a minor allele frequency (MAF) higher than 5%, which limits the number of low-frequency and rare variants evaluated in the region that might contribute to the disease risk as occurs in other complex diseases [44]. Increasing the sample size may further allow the correlation of low frequency alleles with DNAm levels, and increase the statistical power in the differential methylation assessment. Finally, replication analyses would help to elucidate if our results could be extended to other Latin American populations, as regulatory mechanisms are highly dependent on the environment and the genetic variability of the parasite may also contribute to different epigenetic modifications, as it has been previously reported for other parasitic infections [45].

To our knowledge, this is the first study to assess *cis*-mQTLs in chronic Chagas cardiomyopathy patients. Numerous significant interactions between genetic variants and DNAm levels were identified within the only region significantly associated with the disease. Several of these interactions showed also differential methylation in chronic Chagas cardiomyopathy patients, and validated previous findings. These results provide novel functional insights in the genetic background and gene regulation underlying the pathogenesis of the most severe form of Chagas disease.

## Supporting information

S1 Fig Box plots of the mQTLs in linkage disequilibrium (r^2^>0.4) with the GWAS associated variant with chronic Chagas cardiomyopathy. These plots compare the genotypes of the SNPs that form the mQTLs (x-axis) with the specified CpGs and their Mvalues, which are the log_2_-transformed DNA methylation ratio (y-axis). These variants are also in moderate LD with the GWAS leading variant (rs2458298).
(TIF)

S2 Fig Comparison of the transformed significance levels (-log_10_ *p-values*) of the associated SNPs in the previous GWAS with those corresponding to their *cis*-mQTLs. The transformed *p-values* of the mQTL and GWAS analyses were compared for those SNPs that form mQTL with the DNAm position cg22690720 located in the gene region of the *SAC3D1*.
(TIF)

S3 Fig DNA methylation (DNAm) site positions respecting their nearest genes. For the 152 unique DNAm sites identified, their positions were compared with the Human Methylation EPIC manifest from Illumina and were expressed in percentages. According to their position, the DNAm sites can be located in the first exon (1st exon), in the 3’ or 5’ regulatory regions (3’UTR and 5’UTR), in the gene body (body), exons (exonBnd), at 1,500 or 200 to the transcription start site (TSS1500 and TSS200) or intergenic (intergenic).
(TIF)

S1 Table *cis*-mQTLs with SNPs in moderate LD (r^2^>0.4) with the genetic variant associated to the chronic Chagas cardiomyopathy. (DOC)
