# Supplementary figures and images for: GWAS loci associated with Chagas cardiomyopathy influences DNA methylation levels
Source: PLoS Negl Trop Dis. 2021 Oct 29;15(10):e0009874. doi: 10.1371/journal.pntd.0009874 (PMC8580254; doi:10.1371/journal.pntd.0009874)

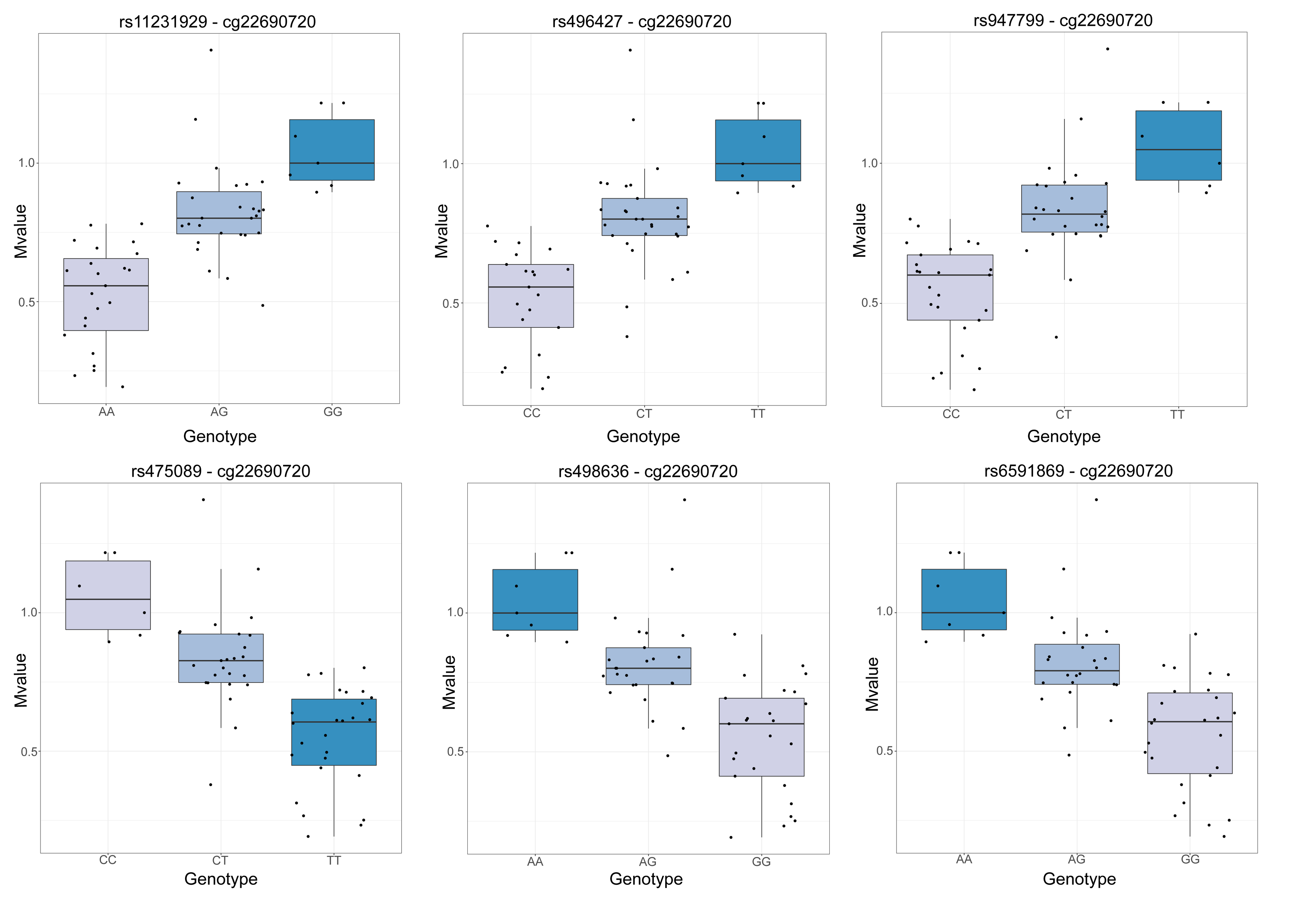

Supplement: S1 Fig — These plots compare the genotypes of the SNPs that form the mQTLs (x-axis) with the specified CpGs and their Mvalues, which are the log2-transformed DNA methylation ratio (y-axis). These variants are also in moderate LD with the GWAS leading variant (rs2458298). (TIF) [file pntd.0009874.s001.tif]

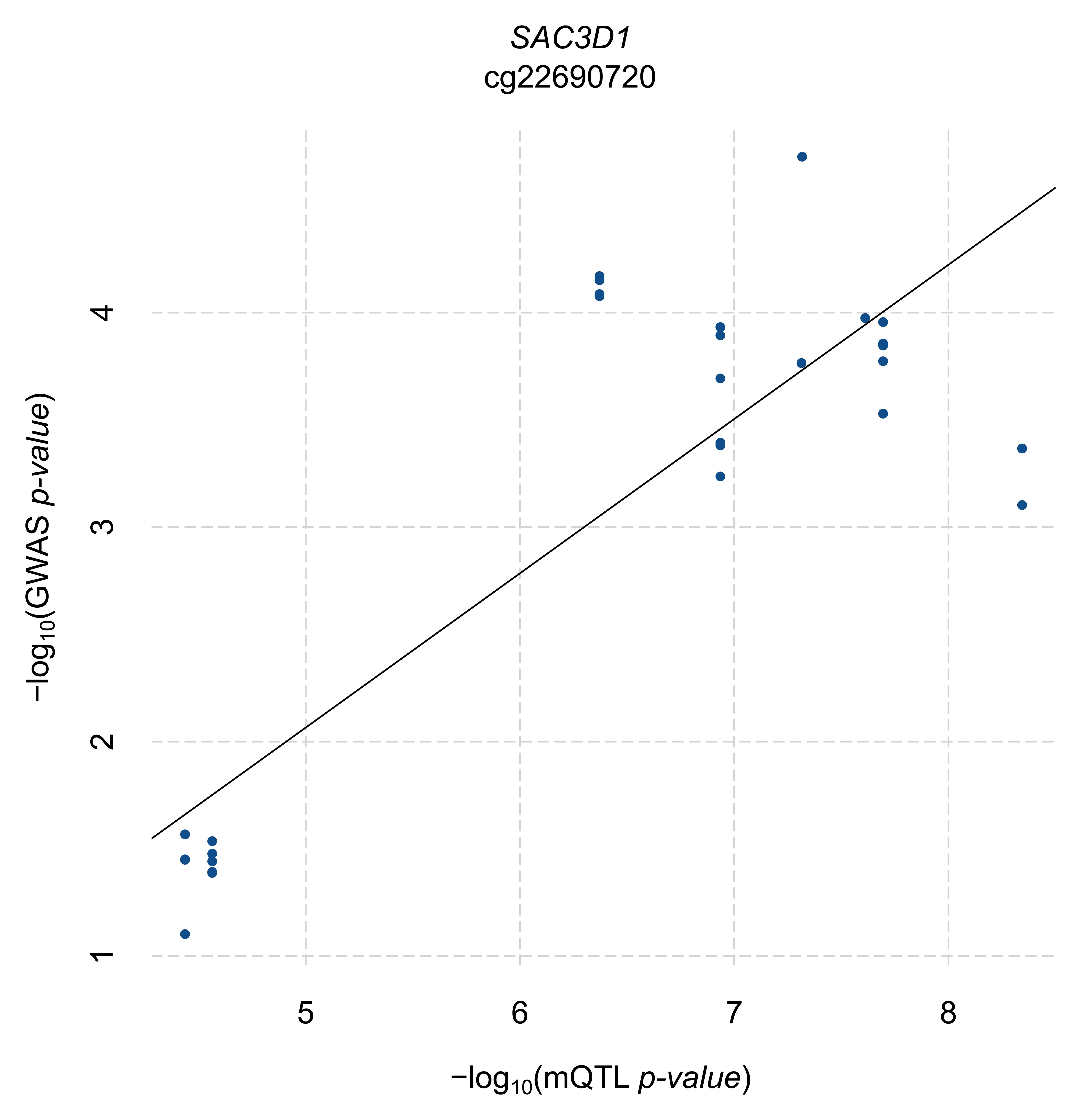

Supplement: S2 Fig — The transformed p-values of the mQTL and GWAS analyses were compared for those SNPs that form mQTL with the DNAm position cg22690720 located in the gene region of the SAC3D1. (TIF) [file pntd.0009874.s002.tif]

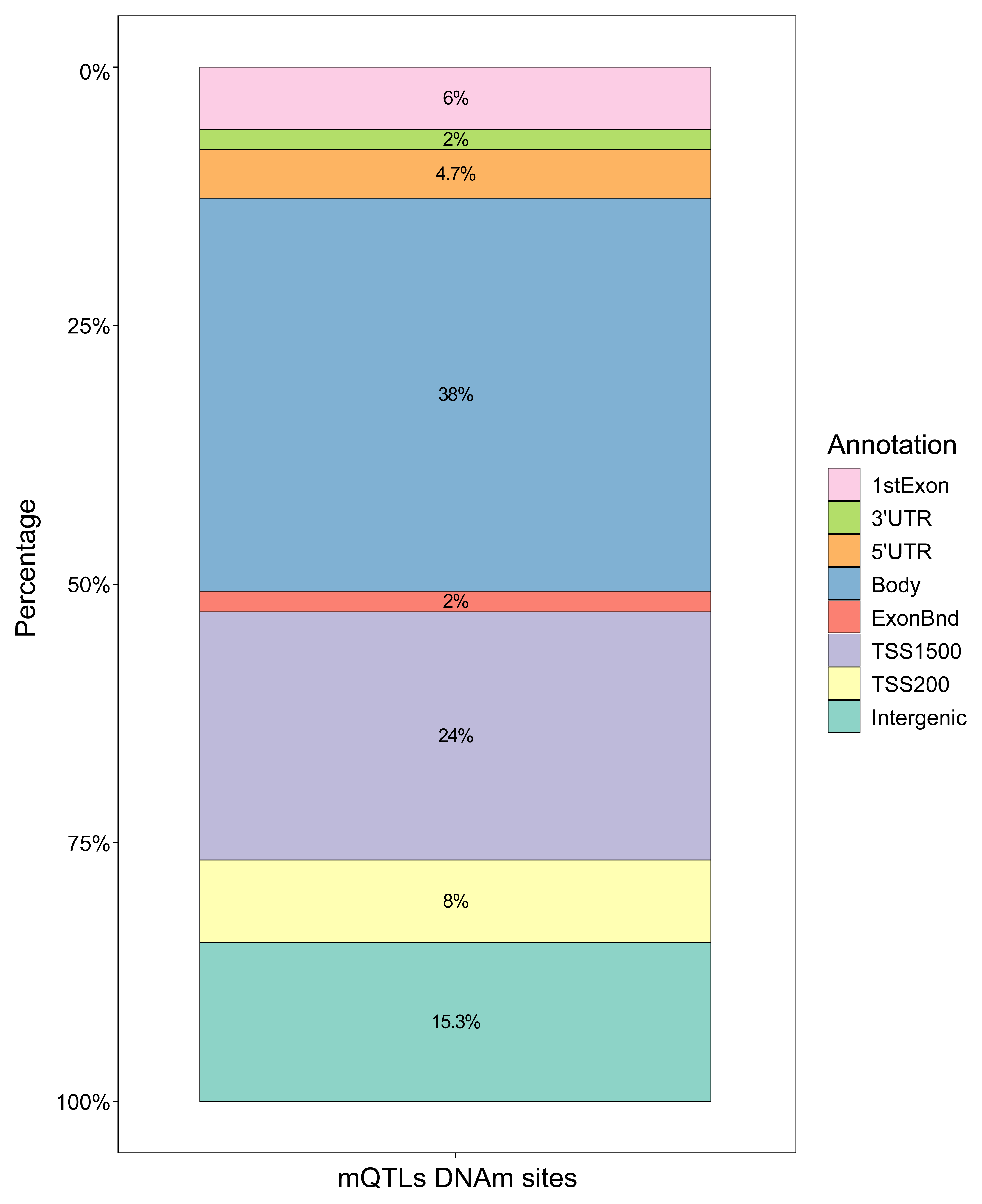

Supplement: S3 Fig — For the 152 unique DNAm sites identified, their positions were compared with the Human Methylation EPIC manifest from Illumina and were expressed in percentages. According to their position, the DNAm sites can be located in the first exon (1st exon), in the 3’ or 5’ regulatory regions (3’UTR and 5’UTR), in the gene body (body), exons (exonBnd), at 1,500 or 200 to the transcription start site (TSS1500 and TSS200) or intergenic (intergenic). (TIF) [file pntd.0009874.s003.tif]
