# Supplementary material for: GWAS loci associated with Chagas cardiomyopathy influences DNA methylation levels
Source: PLoS Negl Trop Dis. 2021 Oct 29;15(10):e0009874. doi: 10.1371/journal.pntd.0009874 (PMC8580254; doi:10.1371/journal.pntd.0009874)
Supplement: S1 Table — (DOC) [file pntd.0009874.s004.doc]

**SUPPLEMENTARY MATERIAL**

| **S1 Table. *cis*-mQTLs with SNPs in moderate LD (r^2^>0.4) with the genetic variant associated to the chronic Chagas cardiomyopathy** | | | | | | | | | | |
| --- | --- | --- | --- | --- | --- | --- | --- | --- | --- | --- |
| **SNP ID** | **SNP position** | **DNAm site ID^a^** | **DNAm position** | **DNAm site function^b^** | | **DNAm site gene** | **FDR** | **Beta** | **R^2c^** | **Distance (bp)^d^** |
| rs11231929 | 11:64810771 | cg22690720 | 11:64808083 | TSS1500 | *SAC3D1* | | 1.85E-05 | 0.26 | 0.49 | 2,688 |
| rs496427 | 11:64838173 |  |  |  |  |  | 6.82E-05 | 0.26 | 0.46 | 30,090 |
| rs947799 | 11:64851305 |  |  |  |  |  | 8.06E-05 | 0.26 | 0.45 | 43,222 |
| rs475089 | 11:64832939 |  |  |  |  |  | 1.46E-04 | 0.26 | 0.44 | 24,856 |
| rs498636 | 11:64807541 |  |  |  |  |  | 3.11E-04 | 0.23 | 0.42 | -542 |
| rs6591869 | 11:64802267 |  |  |  |  |  | 9.49E-04 | 0.22 | 0.39 | -5,816 |
| a DNAm site identificator b Position of the DNAm site respecting its annotated gene. TSS1500 refers a position 1500 bp to the gene promoter. c mQTL correlation. d SNP-DNAm site genetic distance. | | | | | | | | | | |
